# Supplementary material for: Mechanisms governing the pioneering and redistribution capabilities of the non-classical pioneer PU.1
Source: Nat Commun. 2020 Jan 21;11:402. doi: 10.1038/s41467-019-13960-2 (PMC6972792; doi:10.1038/s41467-019-13960-2)
Supplement: Supplementary file 7 — Source data [file 41467_2019_13960_MOESM7_ESM.zip › Source_Data/Figure6/Figure6B_MotifScanOutput/ETS1_mutPU.1/homerResults/motif4.similar.html]

motif4

## Information for motif4

C
T
G
A
T
A
G
C
T
G
C
A
C
A
T
G
A
T
C
G
C
T
G
A
A
G
C
T
C
T
A
G
C
G
A
T
A
C
T
G
C
T
A
G
A
T
C
G
  
Reverse Opposite:  

T
A
G
C
G
A
T
C
T
G
A
C
G
C
T
A
G
A
T
C
C
T
G
A
G
A
C
T
T
A
G
C
G
T
A
C
A
C
G
T
A
T
C
G
G
A
C
T
  

|  |  |
| --- | --- |
| p-value: | 1e-459 |
| log p-value: | -1.058e+03 |
| Information Content per bp: | 1.606 |
| Number of Target Sequences with motif | 2075.0 |
| Percentage of Target Sequences with motif | 15.39% |
| Number of Background Sequences with motif | 1093.0 |
| Percentage of Background Sequences with motif | 3.12% |
| Average Position of motif in Targets | 99.7 +/- 49.9bp |
| Average Position of motif in Background | 99.3 +/- 59.2bp |
| Strand Bias (log2 ratio + to - strand density) | -0.1 |
| Multiplicity (# of sites on avg that occur together) | 1.02 |
| Motif File: | file (matrix) reverse opposite |

### Similar de novo motifs found

|  |  |  |  |  |  |  |  |
| --- | --- | --- | --- | --- | --- | --- | --- |
| Rank | Match Score | Redundant Motif | P-value | log P-value | % of Targets | % of Background | Motif file |
| 1 | 0.841 | A G C T A C G T T C G A A T G C G T A C G C T A A T G C G T C A G A C T A G T C G A T C G A C T | 1e-348 | -801.916102 | 9.13% | 1.23% | motif file (matrix) |
| 2 | 0.753 | C T G A C T A G T G C A C A T G C A T G T G C A A C G T A C T G A C G T C T A G A C T G A C T G | 1e-38 | -88.340194 | 1.84% | 0.52% | motif file (matrix) |
